# Paste

**Published:** 1870-02

**Authors:** 


Paste.—The following is a recipe for making common paste, which will keep for a long time without fermentation: Dissolve an ounce of alum in a quart of water warmed; when cold, add as much flour as will give it the consistency of cream; then strew into it as much powdered rosin as will lie on a five cent nickel, and two or three cloves, ground. It will keep for a year, and when dry may be softened with water.—*Ibid.*

